# The Relation between the Physician and Pharmacist

**Published:** 1872-05

**Authors:** Th. Schumann

**Affiliations:** Atlanta, Georgia; Member of the American Pharmaceutical Association


### THE RELATION BETWEEN THE PHYSICIAN AND PHARMACEUTIST.

BY TH. SCHUMANN, ATLANTA, GEORGIA'
*Member of the American Pharmaceutical Association.*

Audiatur et altera pars!

A report of certain resolutions adopted by the Georgia Medical Society, at Savannah, appearing in the papers, and a private discussion of the same, induces the writer to make a few remarks on certain points in regard to the relation between the physician and the apothecary. Will you please hear the views of one who is by his vocation deeply interested in these questions, and whose profession is naturally associated with your profession in the development of science, and in the practical application of the achievements of science to the benefit of the human race?

By the Georgia Medical Society, in Savannah, a resolution seems to have been adopted to the effect that the druggist should not inform the patient of the nature and composition of a prescription; and if uncertain in regard to anything in the same, should consult the prescribing physician, and not substitute his own prescription for that of the physician.

If, in the State of Georgia, proper regulations existed regarding the practice of medicine and the keeping of drug-stores—or, rather, if the rules and regulations existing were in practical operation—the resolution would not have been necessary; but while as it is, all sorts of persons, not only regular pharmacists, but physicians and others who have no pharmaceutical education, keep drug-stores, all kinds of irregularities may happen.

In ancient times, the savant was priest and physician—gathered roots and herbs, and prepared his draughts himself, as is yet the case with the savages. Since the science progressed to its present point, it is impossible for a man to be perfect in all its branches. Medicine and pharmacy had long ago to be divided into separate departments, and medicine itself seems to be already undergoing the same process of division. Pharmacy—especially chemistry, as a part of it—has acquired such large dimensions, that a man has to be an industrious student to arrive at a tolerable degree of perfection. If a man tries his hand in more than one of these departments, he is apt to become a jack-of-all-trades, and master-of-none; what we properly style a quack.

The regular pharmacist knows his place; he does not wish to transcend his bound; he wishes only to prepare the physicians' prescriptions, and does not care for prescribing himself. But he finds his satisfaction in preparing the medicines in the best manner and of the best material. To this end, he has to study botany, chemistry, materia medica, but not anatomy, pathology, and therapy; and therefore can not, and does not want to, apply those sciences, and can not take the responsibility even in a case of emergency. To interfere with the orders or the duties of a physician, would be assumption on his part. The physician, however, who keeps a drug-store, might easily be induced to consult his own view of a case in question, and might act consequently; and if many drug-stores are kept by physicians, as is the case in Georgia, the medical associations are right to adopt resolutions against the irregularities in question. The pharmacist, however, calls it properly, quackery, if any one keeps a drug-store who is not entitled to it by his pharmaceutic education, and is not at all astonished to see such a druggist physician violate the laws of his own profession. The best guard, therefore, for both the physician and the pharmacist, against quackery of any kind, would be the enforcement of a proper law regulating the practice of medicine and pharmacy respectively, and especially the keeping of drug-stores.

Another resolution says that a druggist should not repeat a prescription without the order of the prescribing physician. It will be very difficult to enforce this regulation in practice, and it is to be feared, therefore, that it would remain a dead law, disregarded by everybody, although no one might deny its utility, and especially its propriety in principle. The physicians seem to be inclined to claim the prescription as their property. The pharmacist does not claim it at all, although he has it in his possession, and possession is, as everybody knows, sometimes the best title. But the question arises, whether it is not the property of the patient. If the physician is called upon by a patient for medical advice, he gives part of it in words, regulating the patient's deportment, diet, and so on—part of it in ordering the medicaments to be used. In most cases, the prescription is the least important part of his advice; and in many cases, the patient has to judge for himself how long he should continue to follow his advice, and to take the medicine. The patient has to judge for himself, or his friends for him, at what moment he needs the physician's advice, and also decides how long he is to continue.

Under all circumstances, it would be very difficult to enforce the rule proposed by this resolution. The courts are not very likely to sustain the claim of the physician. Innumerable disputes would arise, because the druggist would find it very difficult to refuse any of his customers a repetition of their prescriptions; he would also find it very much against his interest to do so. It is not to be seen what the physician could do should the druggists unite in order to oppose his wishes. It has been said the physicians might, in order to enforce the proposed rule, cease to write prescriptions, and rather dispense the required medicines themselves. But the dispensing of medicines by physicians, except in cases of necessity, is surely a conquered stand-point—not practicable, for the following reasons:

1. The dispensing physician would, by this measure, go back to a more primitive state of civilization; he would be compelled to pay more attention to things not strictly belonging to his province, and would be prevented from concentrating his study upon his special profession.

2. It would not be in accord with the great principle of division of labor, and would, therefore, turn out to be an unprofitable business.

3. The dispensing of medicines by the physician is unpopular, especially with the educated classes; it would occupy too much of his time, especially if he is extensively consulted, and would compel him to keep a large stock of medicines on hand.

Instead of bringing the two professions into opposition, it would be to the advantage of all parties concerned, if the public mind could be influenced in the proper manner. The public have to learn that they will always do best to give themselves entirely up to the skillful physician. Well-educated people will do this much easier than uneducated ones, and, therefore, physicians, as well as all other educated men, should advocate education and progress.

In some of the other States of the Union, a great movement is going on, intended to induce the State Legislatures to adopt rules and regulations regarding the keeping of drug-stores, and this movement was chiefly set on foot by the pharmacists themselves. They very properly see that their own interest is best provided for by proper regulations, and that thereby the profession is elevated. Such regulations, provided they be of the proper kind, and be enforced, would guard the public against imposition by quacks—would guard the pharmacist against competition by imposters, and would guard the physician against invasion of his province on the part of the druggist. But until we have such rules and regulations, let the physician protect the regular pharmacist against the medicine vender, who can not be conscientious, because he has no heart for the profession; and let us, meanwhile, agitate the installment of a good law.

The physician and the pharmacist are naturally associated for the purpose of promoting their sciences and the welfare of our race by the application of their attained skill to the suffering. If in each of those sciences, great achievements have been accomplished within the last century, the greater achievement was accomplished by the association of the physiologist and the chemist. The same would take place in practical life, if physicians and pharmacists would divide the labor before them—would keep their provinces separated, and at the same time associate, as they do in other countries, to elevate their respective professions, particularly if aided by appropriate legislation. Both professions would be benefited by it, as well as the public.

